# The role of URO17® in diagnosis and follow up of bladder cancer patients

**DOI:** 10.1186/s12894-024-01426-7

**Published:** 2024-02-09

**Authors:** Mohamed Ibrahim, Joshua Rabinowitz, Rebecca Hilbert, Aruni Ghose, Samita Agarwal, Rajiv Swamy, Ismail Bulut, Mirian Guttierrez, Ebtisam Buali, Ekram Nassar, Parag Jhavar, Fatima Al-Hashimi, Nikhil Vasdev

**Affiliations:** 1 Department of Urology, Lister Hospital, East & North Hertfordshire NHS Trust, Coreys Mill Lane, Stevenage, Hertfordshire UK; 2 Mount Vernon Hospital, Northwood, UK; 3 King Hamad University Hospital, Al Sayh, Bahrain; 4 School of Life and Medical Sciences, University of Hertfordshire, Hatfield, United Kingdom

**Keywords:** URO17®, Bladder cancer, Non-muscle invasive tumor, cancer follow-up, Urothelial cancer

## Abstract

**Objective:**

to evaluate the role of urinary URO17® biomarker in the detection of urothelial tumors in haematuria patients and the detection of recurrence in non-muscle invasive bladder urothelial tumors.

**Materials and methods:**

Our study was formed of two cohorts of patients, group I represents patients presenting with haematuria (*n* = 98), while group II represents patients with known non-muscle invasive bladder cancers on their scheduled follow up cystoscopic investigation (*n* = 51). For both groups, patients were asked to provide urine samples before cystoscopy, either primary as part of the haematuria investigation or as a scheduled follow-up. Urine samples were sent anonymously for standard urine cytology and URO17® biomarker immunostaining. Results were compared to cystoscopic findings using Chi-square analysis and Fisher’s exact test (*P* < 0.05).

**Results:**

Group I was formed of 98 patients, with an average age of 60 years. URO17® showed 100% sensitivity and 96.15% specificity with a negative predictive value (NPV) of 100 and a positive predictive value (PPV) of 95.83. The results showed statistical significance with *P* value < 0.001. Group II was formed of 51 patients, with an average age of 75 years. URO17® was shown to have a sensitivity of 85.71% and NPV of 95.45. Eleven patients of group II were on scheduled BacillusCalmette-Guerin (BCG) and another 5 received Mitomycin C (MMC). The overall results of both groups combined (*n* = 149) showed statistical significance between flexible cystoscopy results and the results of urinary URO17® and urine cytology.

**Conclusion:**

URO17® has a potential to be a reliable test for diagnosis and follow up of urothelial cancer patients and a screening tool adjunct to flexible cystoscopy.

**Trial Registration:**

Not applicable as the current study is not a clinical trial, as per according to the National Institutes of Health, “studies that involve a comparison of methods and that do not evaluate the effect of the interventions on the participant do not meet the NIH clinical trial definition.”

## Introduction

The global annual incidence of bladder cancer is 430,000 with 197,000 among Europe, ranking it the fourth prevalent cancer in men and the fifth in women [1]. With a high rate of recurrence, bladder cancer patients require frequent surveillance, contributing to high financial load for both treatment and follow up [2].

Despite the high prevalence rate, bladder cancer still lacks a screening program in the UK. At the moment, the investigations of bladder cancer depend mostly on a presenting symptom, which is haematuria. The UK National Institute of Health Care and Excellence (NICE) guidance recommends the urgent referral of haematuria patients to the urology department as shown in Table 1 [3].

**Table 1:** NICE Referral guidelines for suspected bladder cancer [3]

Age criteria	Symptom criteria
Aged 45 and over	Unexplained visible hematuria without urinary tract infection.
Aged 45 and over	Visible hematuria that persists or recurs after successful treatment of urinary tract infection.
Aged 60 and over	Unexplained non-visible hematuria and either dysuria or a raised white cell count on a blood test.

Having an odds ratio of 34, haematuria is a significant predictor of bladder cancer. However, the malignancy detection rate in haematuria patients is 18.9%, making this symptom neither sensitive nor specific for cancer [4]. Nearly half of bladder cancer patients present with nonspecific symptoms including abdominal pain, constipation, or urinary tract infection. The other half of bladder cancer patients present with visible haematuria [5]. Accordingly, improvement of diagnostic methods, particularly for the nonvisible haematuria bladder cancer patients, is needed [6].

Cystoscopy is the gold standard diagnostic tool to diagnose bladder cancers as it allows proper assessment of the bladder and urethral urothelial lining. However, its invasiveness is bothersome for the patients and cannot assess the upper tracts [7]. Consequently, upper urinary tracts are investigated by radiological scans such as CT urography. Both cystoscopy and CT urography are indicated for all urologically referred haematuria patients during the first urological review, exposing patients to loads of unnecessary invasive diagnostic procedures and radiation [8, 9]. Costing around £100 million a year in the UK alone, these unnecessary investigations are also an economic burden [10, 11].

With the progression in bladder neoplasia molecular biology research, genetic markers have been detected in tumor development and progression [12]. This research progression led to the detection of some markers in the urine of bladder cancer patients. Urine samples are easy to collect and have the advantage of direct contact with the urothelial tumours [13].

Keratin 17 (K17) is one of the cytokeratin proteins expressed normally in nail beds and hair follicles [14]. K17 is present in stem cells of ectoderm, such as skin appendages and endocervical mucosa. It is absent in normal mature epithelia and is re-expressed in cancers [15–21]. The association of K17 as an oncoprotein with poor prognosis in multiple cancers as cervical, esophageal, lung and bladder cancer has been reported in previous studies [22–24]. This study aimed to evaluate the role of urinary URO17® biomarker in the detection of recurrence of non-muscle invasive bladder urothelial tumors.

## Materials and methods

### Study design

**Fig. 1:**
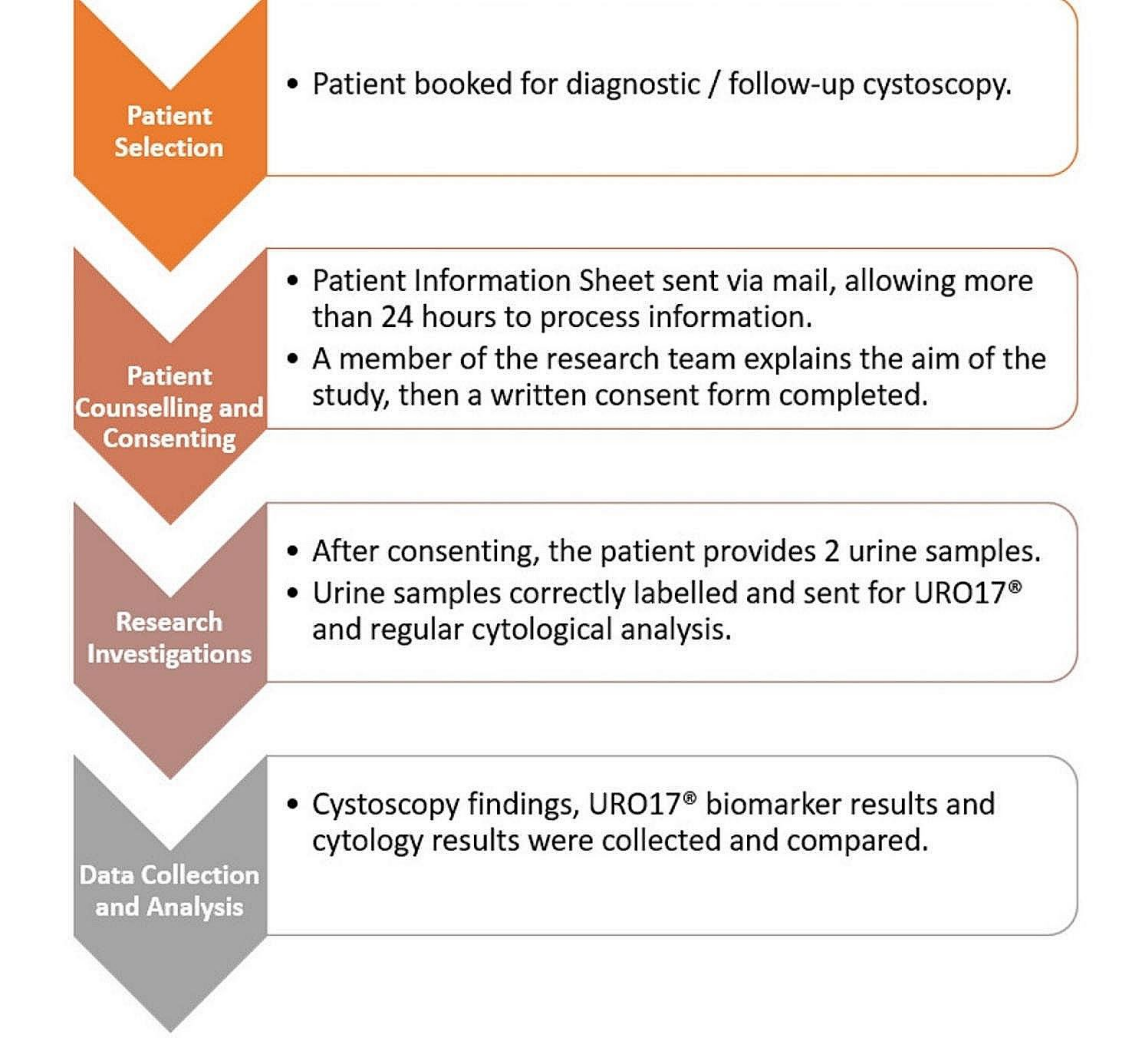
Study design flowchart

This study is a prospective blinded validation trial with two cohorts of patients. Group I consisted of 98 patients presenting with haematuria, and group II consisted of 51 patients with known NMIBC and upper tract urothelial cancers, who are under scheduled follow up cystoscopy. The study was multicentric, performed at East and North Hertfordshire NHS Trust in the United Kingdom and King Hamad University Hospital in Bahrain (KHUH). Urologists, pathologists and patient advocates participated in the study. The study design is demonstrated in Fig. 1. Inclusion and exclusion criteria of patients recruited in each group are shown in Table 2.

**Table 2:** Inclusion and exclusion criteria per group

	Group I	Group II
Inclusion criteria	- Patients aged ≥ 18 years of age - Patients with visible or microscopic haematuria	- Age > 18 - Non-muscle invasive bladder cancer and upper tract urothelial cancer patients on regular follow up
Exclusion criteria	- Patients aged < 18 years of age - Patients with catheter in situ - Patients who are currently undergoing radiation therapy - Patients currently on investigational drug trials. - Patients refused to participate or lack the capacity to sign the consent	- Age < 18 - Muscle-invasive bladder cancer patients - Patients currently on investigational drug trials - Patients refused to participate or lack the capacity to sign the consent

### Ethics approval

Local ethics approval for the current study was obtained through the research and development departmental procedure. IRAS Application 253,585 submitted and national ethics/protocol approval received (18/EE/0395) through NHS Health Research Authority (HRA) Research Ethics Committee. Informed consent to participate was obtained from all the participants in the study.

### Data collection

Following patients consenting, two urine samples per participant were collected on the day of cystoscopy and correctly labelled, one sent for normal urine cytology and the other for urinary URO17® biomarker. Regarding group II, primary histological diagnosis, age and previous treatment with BacillusCalmette-Guerin (BCG) or Mitomycin C (MMC) were noted. Cystoscopy findings, which are considered the gold standard for detecting tumor or tumor recurrence, were added to the data [7].

### Urine sample preparation

After anonymization of urine samples, they were stored at 4 °C, then centrifuged for 5 min on a Sorvall ST 16 Centrifuge (Thermo Scientific) at 1500 r.p.m. The pelleted cell deposits were inspected and the supernatant was disposed. When deposits weren’t visible, a vial of PreservCyt fluid (Hologic) was placed into the Universal container, then the contents were mixed and tipped back into the PreservCyt vial.

When the deposit amount was large but not bloodstained abundantly, the deposit was stirred using a Pasteur pipette, then three drops were applied to the PreservCyt vial. When the deposit was abundantly stained with blood, 15 mL of CytoLyt fluid (Hologic) was added to lyse the blood in the deposit, then centrifuged for 10 min at 1500 r.p.m. then 3 drops were applied to the PreservCyt vial.

Using non-gynecological blue filters, samples were processed on an automated ThimPrep 2000 processor (Hologic). A ThinPrep slide with a monolayer of cells is the result of this process. Then, the slides were fixed with Cytofix spray (Hologic) and allowed to dry for 15 min. Sample slides were stored between 2 and 8 °C and stained in weekly batches using URO17® (Lot: A8034124, 3.42 mg/mL, 1:5000 dilution in 10% CS) on a Leica detection kit DS9800 for 20 min.

### Diagnostic test evaluation

The URO17® staining results were analysed by blinded pathologists and results scored as per scoring system in Table 3.

**Table 3:** URO17® pathology scoring system

Score	Staining
0 —Negative	No stained cells present
1—Negative	Weak staining (1+)
2—Positive	Strong staining (2 + and above) > 20 positive staining of cells based on staining intensity.

### Statistical analysis

Data were statistically analysed using a software program (IBM SPSS Statistics v23; IBM Corp, New York, USA), with statistical significance set at *P* < 0.05. Qualitative data were described using number and percentage. The sensitivity, specificity, positive predictive value (PPV), negative predictive value (NPV), and accuracy of the data were calculated. The Chi-square test was used to compare between different groups for categorical variables, and the Fisher’s exact test was used for correction of Chi-Square test.

## Results

This study involved 149 participants, 93 male and 56 female, with average age of 65 years. Group I consisted of 98 patients presenting with haematuria, and group II consisted of 51 patients with known NMIBC and upper tract urothelial TCC, who are under scheduled follow up cystoscopy. Urinary URO17® and urine cytology results were compared to flexible cystoscopy results in both groups and displayed in Table 4 for group I and Table 5 for group II.

**Table 4:** Group I (*n* = 98): Sensitivity, specificity and accuracy for urinary URO17® and urine cytology

	Cystoscopy				Sensitivity	Specificity	PPV	NPV	Accuracy
	Free of malignancy (*n* = 52)		Positive of malignancy (*n* = 46)						
	No.	%	No.	%					
Urinary URO17®									
Negative	50	96.0	0	0.0	100.0	96.15	95.83	100.0	97.96
Positive for malignancy	2	3.8	46	100.0					
χ^2^ (*P*)	90.304^*^ (< 0.001^*^)								
Urine cytology									
Inflammatory	26	50.0	4	8.7	91.30	50.0	61.76	86.67	69.39
Positive for malignancy	26	50.0	42	91.3					
χ^2^ (*P*)	19.604^*^ (< 0.001^*^)								

χ^2^: Chi square test MC: Monte Carlo

*: Statistically significant at *P* ≤ 0.05

**Table 5:** Group II (*n* = 51): Sensitivity, specificity and accuracy for urinary URO17® and urine cytology

	Cystoscopy				Sensitivity	Specificity	PPV	NPV	Accuracy
	Free of malignancy (*n* = 44)		Recurrence of malignancy (*n* = 7)						
	No.	%	No.	%					
Urinary URO17®									
Negative	21	47.7	1	14.3	85.71	47.73	20.69	95.45	52.94
Positive for malignancy	23	52.3	6	85.7					
χ^2^ (^FE^*P*)	2.753 (0.124)								
Urine cytology									
Inflammatory	41	93.2	5	71.4	28.57	93.57	40.0	89.13	84.31
Positive for malignancy	3	6.8	2	28.6					
χ^2^ (^FE^*P*)	3.232 (0.133)								

χ^2^: Chi square test FE: Fisher Exact

*: Statistically significant at *P* ≤ 0.05

In group I (*n* = 98), URO17® showed 100% sensitivity and 96.15% specificity with a NPV of 100 and a PPV of 95.83. The results showed statistical significance with P value < 0.001 as shown in Table 4.

In group II (*n* = 51), URO17® showed 85.71% sensitivity and 47.73% specificity. It revealed a NPV of 95.45 and a PPV of 20.69. There was no statistical significance as shown in Table 5. Interestingly, among the patients with false positive results (*n* = 23), 16 patients had previously received BCG or MMC, which implies that these therapies may contribute to false positive results for URO17®. In BCG, the reason for false positive results is due to the nature of K17 being a regenerative protein. Therefore, when the cells are damaged by BCG, these cells express more K17 to regenerate and repair.

Regarding the primary histopathology of group II (*n* = 51), 29 (56.86%) patients had pTa and 18 (35.29%) patients had pT1 non-muscle invasive bladder cancer. The remaining 4 (7.84%) patients had upper urothelial TCC as shown in Table 6.

**Table 6:** Prevalence of primary pathological diagnosis in group II (*n* = 51)

Primary pathology	Number of patients
PTa	29 (56.86%)
pT1	18 (35.29%)
Upper tract urothelial tumors	4 (7.84%)

Overall results regarding URO17® within all participants (*n* = 149) showed sensitivity of 98.11%, specificity of 73.96%, PPV of 67.53 and NPV 98.61, showing statistical significance as shown in Table 7. Overall urine cytology results showed 83.02% sensitivity and 69.79% specificity. It revealed a NPV of 88.16 and a PPV of 60.27. There is statistical significance between flexible cystoscopy results and the results of Urinary URO17® and urine cytology as shown in Table 7 within both groups.

**Table 7:** Overall results for groups I and II

	Cystoscopy				Sensitivity	Specificity	PPV	NPV	Accuracy
	Free of malignancy (*n* = 96)		Positive of malignancy (*n* = 53)						
	No.	%	No.	%					
Urinary URO17									
Negative	71	74.0	1	1.9	98.11	73.96	67.53	98.61	82.55
Positive for malignancy	25	26.0	52	98.1					
χ^2^ (*P*)	71.029^*^ (< 0.001^*^)								
Urine cytology									
Inflammatory	67	69.8	9	17.0	83.02	69.79	60.27	88.16	74.50
Positive for malignancy	29	30.2	44	83.0					
χ^2^ (*P*)	38.110 (< 0.001^*^)								

χ^2^: Chi square test MC: Monte Carlo

*: Statistically significant at *P* ≤ 0.05

## Discussion

With a negative predictive value of 98.61, URO17® urinary test is a potential non-invasive diagnostic and follow up test for bladder and urothelial tumors of the upper tract which spares patients the radiological hazards of CT scans or the invasiveness of cystoscopy.

Results of the current study conveyed significantly higher sensitivity of URO17® for detection of urothelial cancers in comparison to other urinary biomarkers. In 2015, Chou et al. performed a meta-analysis on a nuclear matrix protein (NMP), NMP22. NMP22 bladder cancer ELISA-Test, a quantitative test, and NMP22 BladderChek test, a qualitative test, have been approved by the US Food and Drug Administration. Quantitative ELISA test showed a sensitivity of 69% and a specificity of 77%, while the qualitative test showed 58% for sensitivity and 88% for specificity [25–27].

Another liquid urinary biomarker test, UroVysion, is a multicolour fluorescent in situ hybridisation assay (FISH) which spots chromosomes 3, 7, or 17 aneuploidy or loss of the 9p21 locus. The sensitivity of UroVysion is between 69% and 87% with specificity ranging from 89 to 96% [25].

These results support those of previous studies of URO17® regarding high sensitivity. However, in contrast to previous research, our study reported a lower specificity of 73.96% [13]. This might be due to that our patients’ cohort included patients who have received BCG or MMC treatments, which led to higher false positive staining with URO17®.

Since this study required rigid cystoscopy and resection for histological diagnosis, for cystoscopy positive participants, patients must have been seen by urologists. Their histopathology was then discussed in our MDT meeting for optimum categorization.

To overcome the potential problem of visible haematuria affecting proper staining and analysis of the urine sample, CytoLyt fluid was used in slide preparation. The high sensitivity of biomarkers results in “anticipatory positive results” prior to being truly visible cystoscopically [28].

Regarding its clinical application, urinary URO17® biomarker has the potential to be a cost effective and non-invasive diagnostic test for urothelial carcinoma with high sensitivity and negative predictive value. According to NICE innovation briefing, the cost of the URO17® test is £110 per test (excluding VAT) and that of flexible cystoscopy ranges from £229 to £258 [29]. Additionally, URO17® urinary biomarker test can be adjuvant to regular follow-up check cystoscopy for early detection of recurrences.

URO17® test is an immunocytochemical assay that uses the same urinary samples collected for normal urine cytology, unlike other more elaborate and expensive urinary biomarker assays. Those pathologists familiar with urine cytology analysis will not find it difficult to interpret URO17®, allowing for a more efficient workflow. Moreover, the laboratory setup is almost the same, requiring no extra instrumentations, which makes the test widely applicable.

Although it might be argued that URO17® is just another biomarker among numerous non-invasive investigations, this study highlighted the use of URO17® in diagnosis and follow up of urothelial cancer patients and as a potential screening tool. This confirms the findings of Babu et al. and Vasdev et al. regarding the reliability of URO17® testing.

Data of the current study imply that URO17® testing may be used for detection of recurrent papillary and nonpapillary carcinomas of the bladder. Our cohort also included patients with urothelial tumors in upper tracts, which even cystoscopies failed to detect, making it a more inclusive and comprehensive test. Attributable to its high sensitivity, URO17® urine test can be an initial test during assessment of haematuria patients as well as a regular test for NMIBC patients during their follow-up, sparing cystoscopic and radiological investigations for URO17® positive patients only. A limitation of the current study is the subjectiveness of the flexible cystoscopy test due to being operator-dependant. Further research towards improving and simplifying diagnosis and follow up of urothelial cancer patients is required due to the potential of our initial results.

## Conclusions

URO17® has the potential to be a reliable test for diagnosis and follow up of urothelial cancer patients and a screening tool adjunct to flexible XXXystoscopy. The most conclusive investigation may be provided by combining several biomarkers, with URO17® playing a significant role.

## Data Availability

The raw data of the study is available.
